# A Case of Osmotic Demyelination Syndrome in a Chronic Alcoholic With Moderate Hyponatremia

**DOI:** 10.7759/cureus.15129

**Published:** 2021-05-19

**Authors:** Ibiyemi O Oke, Waneeza Mughees, Hinal Patel, Olubunmi Oladunjoye, Eugene York

**Affiliations:** 1 Internal Medicine, Reading Hospital - Tower Health, West Reading, USA; 2 Internal Medicine, Drexel University College of Medicine, Philadelphia, USA

**Keywords:** osmotic demyelination syndrome, moderate hyponatremia, alcohol use disorder, paraparesis, encephalopathy

## Abstract

Osmotic demyelination syndrome (ODS) is a clinical syndrome seen following aggressive correction of severe hyponatremia. Chronic alcohol use, malnutrition, and electrolyte derangement are additional risk factors promoting the demyelination in ODS. A 49-year-old female with a history of untreated mood disorder, hypertension, alcohol, and tobacco abuse presented to the emergency department (ED) with a three-month history of generalized body weakness. She also had a history of recurrent falls, difficulty walking, inadequate food and water intake, progressively worsening jaundice, and confusion which started about the same time. Her vital signs were normal; some of the significant physical examination findings were: sclera icterus, abdominal distension, bilateral pedal edema, hand tremors, rotary nystagmus, paraparesis, 1+ bilateral knee jerk, and absent bilateral ankle jerk. She had moderate hyponatremia, mild hypokalemia, deranged liver function test with a cholestatic pattern and transaminitis, hypoalbuminemia, elevated ammonia, lipase, in keeping with alcoholic liver disease and acute pancreatitis. In the ED, she received a normal saline infusion, and her serum sodium rose by just 6 mmol/L within the first 24 hours. She had drainage of her ascitic fluid and treatment with thiamine, folic acid, prednisone, lactulose, rifaximin, furosemide, spironolactone, and Ceftriaxone with improvement in clinical and laboratory abnormalities. Her lower extremity weakness persisted despite physical therapy, prompting neurologic evaluation. MRI of the lumbar spine showed an old compression fracture and lumbar spinal stenosis, while MRI brain findings were consistent with Osmotic demyelination. At the time of discharge to a rehabilitation facility, her serum sodium was 132 mmol/L, but her leg weakness persisted. Although rare, ODS can occur in the setting of moderate hyponatremia if there are additional risk factors that lower the threshold for demyelination.

## Introduction

Osmotic demyelination syndrome (ODS) is a rare, preventable, and potentially fatal complication of rapid correction of hyponatremia. It is a non-inflammatory demyelinating disease previously referred to as central pontine demyelination (CPM) because the pons was thought to be the only part of the nervous system affected. ODS is predominantly seen in patients with poor nutritional status and alcoholics [1], but it can also be seen in patients with psychogenic polydipsia, immunodeficiency states, and even following surgical procedures [2,3]. ODS is characterized by loss of myelin sheath with relative preservation of axons and neurons [4]. The central part of the basal pons is the most common site of demyelination in ODS due to the susceptibility of this site to metabolic insult [5]. The availability of brain MRI has increased the number of cases because some at-risk patients are asymptomatic [6]. The most common presentation is encephalopathy [7], but it may be characterized by dysphagia, dysarthria, pseudobulbar palsy, quadriparesis, dystonia, Parkinsonism, and locked-in syndrome [6,8].

Here, we present an unusual case of ODS in a 49-year-old woman with a history of alcohol use disorder who was admitted with moderate hyponatremia, electrolyte disturbance, acute alcoholic hepatitis, and lower limb weakness. 

## Case presentation

A 49-year-old female with a history of untreated mood disorder, hypertension, alcohol, and tobacco abuse; presented to the emergency department (ED) with a three-month history of generalized body weakness. She also had a history of confusion, inadequate food and water intake, and progressively worsening jaundice. Her boyfriend reported she had been lying on the couch for several weeks, and she crawled to the bathroom as she was unable to ambulate due to unsteadiness, dizziness, and lower limb weakness. He reported that the patient chews Cannabidiol gum (CBD) and drinks one liter of rum and two cans of beer daily.

Her vitals in the ED were heart rate 93 bpm, respiratory rate 17 cpm, temp 36.6 degree C, SPO2 94%, BP 101/74 mmHg. Significant physical examination findings were scleral icterus, 4+ bilateral pitting pedal edema, abdominal distention with demonstrable ascites by fluid wave, bilateral intention tremors, rotary nystagmus, power 3/5 in both lower extremities, 1+ bilateral knee jerk, and absent ankle jerk bilaterally. Significant laboratory findings include moderate hyponatremia, hypokalemia, deranged liver function test, leukocytosis, and elevated lipase levels and ammonia (Table 1). Abdominal ultrasound showed mild hepatomegaly with diffuse heterogenicity. Non-contrast CT of the brain and cervical spine were unremarkable. She received 100 ml/hour of normal saline infusion for five hours; a repeat serum sodium dropped to 124 mmol/L. The normal saline infusion was continued for 16 hours with an improvement in serum sodium to 130 meq/l within 24 hours of presentation. This patient had drainage of ascitic fluid and was treated with thiamine, folic acid, prednisone, lactulose, rifaximin, furosemide, spironolactone, and ceftriaxone with improvement in clinical and laboratory abnormalities.

**Table 1:** Laboratory results. WBC: white blood cell; INR: international normalized ratio.

Laboratory test (units)	Result	Normal range
Sodium (mmol/L)	126	136-145
Potassium (mmol/L)	3.0	3.5-5.1
Chloride (mmol/L)	73	98-107
Alkaline phosphatase (IU/L)	584	34-104
Gamma-glutaryltransferase (mg/dL)	903	0.3-1.0
Alanine transaminase (IU/L)	106	7.0-52
Aspartate transaminase (IU/L)	267	13-39
Total protein (g/dL)	6.9	6.4-8.9
Albumin (g/dL)	3.1	3.5-5.7
Ammonia (micromol/L)	56	16-53
Lipase (IU/L)	272	11-82
Direct bilirubin (mg/dL)	16	0.0-0.2
Total bilirubin (mg/dL)	22.2	0.3-1.0
WBC (x10^9^/L)	15.2	4.8-10.8
Lactic acid (mmol/L)	9.5	0.4-1.4
INR	1.5	0.9-1.1
Serum osmolality (mOsm/kg)	275	280-290
Urine osmolality (mOsm/kg)	380	300-1200
Urine sodium (mmol/L)	35	>20

**Figure 1:**
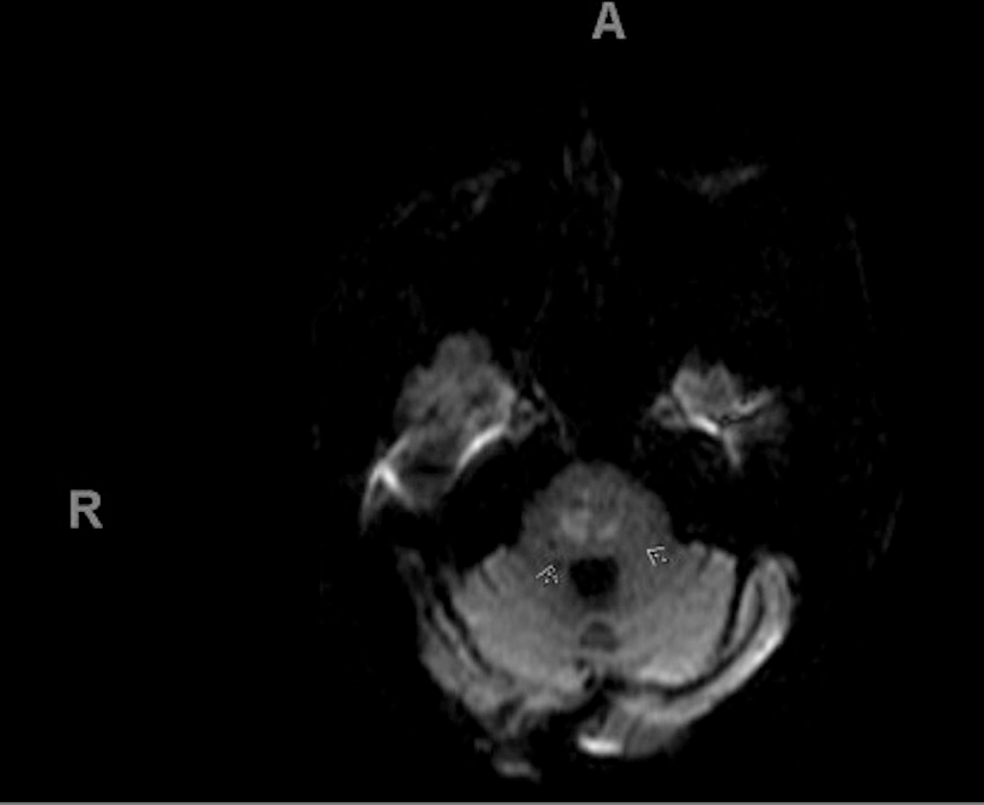
T2-weighted MRI of the brain, arrows showing hyperintense lesions in the central portion of the pons.

**Figure 2:**
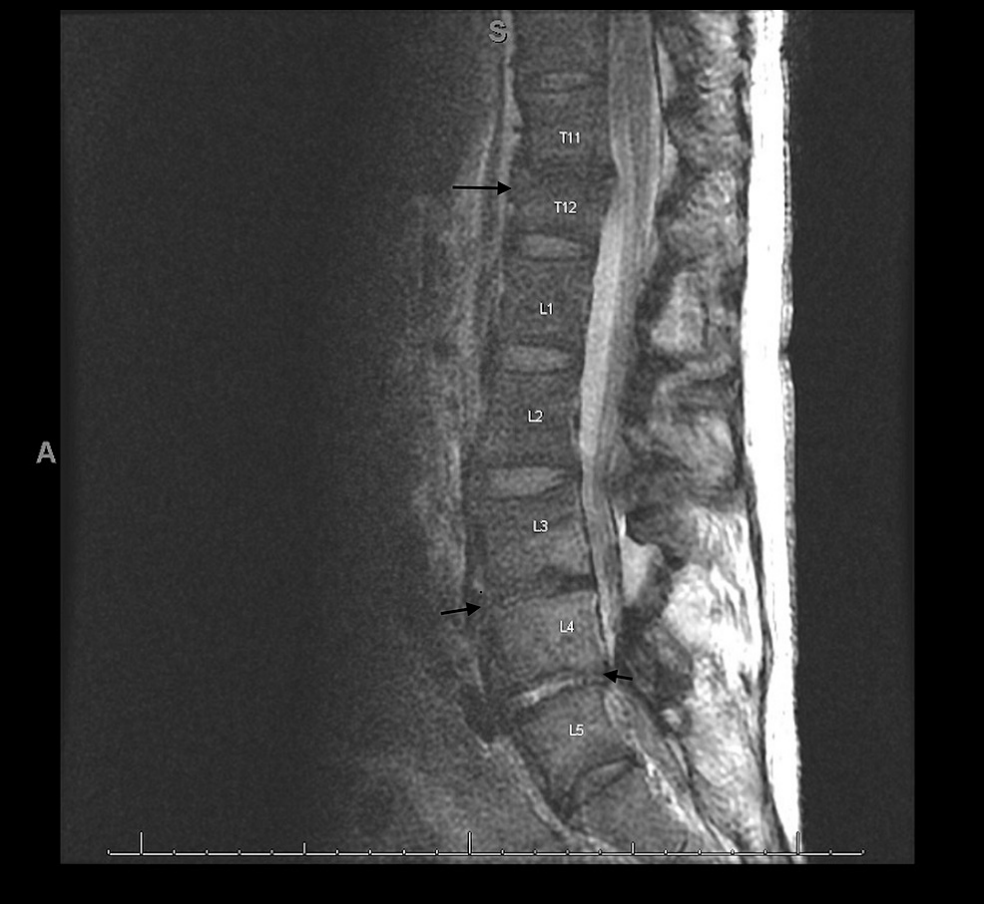
Lumbar spine MRI showing chronic T12 compression deformity, mild spinal stenosis, multifactorial central and left lateral L3/L4 and L4/L5 spinal stenosis, high grade left lateral L4/L5 foraminal stenosis.

Her lower limb weakness at presentation was attributed to deconditioning and possible alcohol myopathy, but her weakness persisted despite three weeks of physical therapy, necessitating further neurologic workup. Non-contrast brain MRI showed areas of hyperintensity within the posterior pons consistent with osmotic demyelination (Figure 1), Lumbar spine MRI showed chronic T12 compression deformity, mild spinal stenosis, multifactorial central and left lateral L3/L4 and L4/L5 spinal stenosis, high grade left lateral L4/L5 foraminal stenosis (Figure 2). She declined electromyography (EMG) but had normal Thyroid-stimulating hormone, Creatine Kinase, and Vitamin B12 level. The lowest serum sodium during hospitalization was 124 mmol/L, and the maximum rate of correction was 6 units in 24 hours. Her hyponatremia was attributed to hypovolemic hypoosmolar hyponatremia from poor oral intake, with some contribution from chronic alcohol use. At the time of discharge to a rehabilitation facility, her serum sodium was 132 mmol/L, but her leg weakness persisted.

## Discussion

ODS encompasses central pontine myelinolysis (CPM) and extra pontine myelinolysis (EPM). Extrapontine myelinolysis can occur in the basal ganglia, cerebral white matter, thalamus, or hippocampus, but it shares the exact pathogenesis as CPM, and both sometimes co-exist in patients [6]. A large autopsy study reports the prevalence of ODS to be 0.25%-0.5% in the general population [9]. Osmotic demyelination is often attributed to rapid correction of chronic, severe hyponatremia [6,10], but our patient only had moderate hyponatremia. As with this patient, ODS can be seen in patients with a less aggressive correction of hyponatremia [6] if there are additional risk factors [7]. Chronic alcohol use likely played a role in this case; it can independently increase myelin damage by interfering with sodium and water regulation via ADH suppression [11]. ODS can arise during the terminal stage of binge drinking in patients with alcohol use disorder and sometimes after alcohol withdrawal [12] due to the osmotic changes resulting from reduced food and water intake during these times. This mechanism aptly describes one of the possible ways our patient developed ODS.

The cause of her encephalopathy is likely multifactorial: ODS, hepatic encephalopathy, alcohol intoxication, and possibly infection. ODS can present with paraparesis, but her lower limb weakness was attributed to deconditioning and alcohol myopathy. Given only moderate hyponatremia, neurologic complications are rare in patients whose serum sodium rise is less than 12 mmol per liter per day [10]; this likely resulted in delayed clinical suspicion of ODS. In addition, she had hypokalemia, which along with other metabolic abnormalities such as hyperglycemia, azotemia, hypophosphatemia, and hyperammonemia, may increase the risk of ODS [11,13,14,15]. 

MRI is the best imaging modality for demonstrating the lesions of ODS. Diffusion-weighted imaging (DWI) is more sensitive than conventional sequencing in demonstrating the lesions but may be negative early in the course of the disease and should be repeated after two to four weeks if there is high suspicion [14]. There have been reports of central pontine abnormalities on MRI of asymptomatic alcoholic men [16], and MRI changes in ODS can persist for up to two years [6]. It is difficult to establish the onset of the neurologic insult in this patient because she presented several weeks after her symptoms started. Her serum sodium did not go below 120 mmol/L during her hospitalization, and there was no aggressive correction of her hyponatremia. Also, she had her brain MRI after one month of being hospitalized. We suspect she developed ODS before presenting to the ED, probably when she first noticed gait disturbance and lower extremity weakness. Although she had spinal stenosis on lumbar spine MRI, she did not report back pain; her radiologic findings did not explain the persistent weakness and inadequate response to physical therapy. She declined electromyography (EMG) and further neurologic workup.

There is still no consensus on the appropriate treatment of ODS. Dopamine agonists and other anti-dystonic medications showed promise in some patients with extrapyramidal features [17]. Re-induction of hyponatremia with desmopressin or intravenous 5% dextrose solution or both have been suggested for patients with low to moderate risk of ODS [18]. Corticosteroid, minocycline, plasmapheresis, and intravenous immunoglobulin have also been tried [9,19,20]. Non-pharmacologic treatment with physical therapy [9] was the treatment modality employed in this patient who developed ODS at an unknown time. Overall, prevention is still the best treatment, and the prognosis of ODS is independent of the severity of neurologic presentation or hyponatremia. Full clinical recovery is possible, but most patients who survive live with neurologic deficits.

## Conclusions

Osmotic demyelination syndrome commonly occurs in the setting of severe hyponatremia. Our patient had neuroimaging and clinical findings in keeping with ODS despite her serum sodium of 126 mmol/L. Unlike most cases of ODS, her hyponatremia was not aggressively corrected, but she had other likely triggers. Physicians should be aware of patients with additional risk factors that may precipitate ODS at a higher level of serum sodium and without rapid correction, as we described in this case. These groups of patients are easily missed and may not benefit from the available treatment options.
